# Prognostic value of microRNAs in head and neck cancers: a systematic review and meta-analysis protocol

**DOI:** 10.1186/s13643-018-0812-8

**Published:** 2018-10-02

**Authors:** Chellan Kumarasamy, Arikketh Devi, Rama Jayaraj

**Affiliations:** 1 School of Bioengineering, SRM University, Kattankulathur, Tamil Nadu 603203 India; 2 Charles Darwin University, Ellengowan Dr, Casuarina, NT 0810 Australia; 3 Charles Darwin University, Yellow 1.1.05, Ellengowan Drive, Darwin, NT 0909 Australia

**Keywords:** Head and neck cancer (HNC), miRNA, Prognosis, Survival, Systematic review, Meta-analysis, Protocol, PRISMA

## Abstract

**Background:**

Head and neck cancers form a significant share of all cancer incidences worldwide. Though treatment modalities exist, post-treatment recurrence and survival rates in recurrent patients continue to be high. MiRNAs offer an effective method of estimating the probability of recurrence and survival of HNC patients, thereby allowing for effective treatment and better survival rates.

**Methods:**

The systematic review protocol was prepared according to the Preferred Reporting Items for Systematic Review and Meta-Analyses Protocols (PRISMA-P) 2015 statement. Relevant studies will be identified by a rigorous search of multiple bibliographical databases, including MEDLINE, Scopus, PubMed, Web of Science, Embase and Science Direct, without any language restrictions (up to June 2018). The primary screening will be performed by a review team via analysis of titles and abstracts of published articles. Final selection of articles will be achieved by two independent reviewers, based on predefined selection criteria. Data will be extracted from eligible studies using a pre-piloted data extraction form. Statistical analysis will be performed on the basis of available data, extracted from eligible studies. Meta-analysis will be performed, and a forest plot will be generated, to determine pooled hazard ratios (HR) and 95% confidence interval (CI) using CMA. A fixed or random-effects model of meta-analysis will be used depending upon the between-study heterogeneity; publication bias will be determined by the Egger’s bias indicator test. A narrative synthesis will be undertaken where statistical data is found to be insufficient.

**Discussion:**

There is a lack of highly sensitive and specific biomarkers for estimating the HNC patients’ prognostic outcomes, particularly in post-treatment conditions. This systematic review will identify and validate specific miRNA as prognostic biomarkers by utilising a collection of previously published data on miRNA expression and survival. Highlighting these prognostic specific miRNAs will have major clinical implications by allowing for better overall treatment strategies and patient survival estimates, by offering clinicians a method of quantitatively analysing prognosis via miRNA expression.

**Systematic review registration:**

This review protocol was registered on PROSPERO and assigned the registration number CRD42017077411.

## Background

Head and neck cancer (HNC) is the sixth leading cancer worldwide, with an annual incidence rate of more than 550,000 cases with around 300,000 deaths each year [1]. It has strong associations with specific environmental and lifestyle risk factors, such as tobacco and alcohol consumption, Epstein-Barr virus (EBV) and human papillomavirus (HPV) infection [2]. Fifty to sixty percent of HNC patients with advanced disease stages have been shown to develop loco-regional recurrences within a 2-year period frequently. Twenty to thirty percent of the HNC patients with recurrences also develop distant metastasis, leading to poor prognosis [3]. HNC diagnosis is based on established diagnostic methods such as histology analysis of biopsy and fusion positron emission tomography-computerised tomography (PET-CT) [4]. Treatment of HNC has also been well established and is of the multimodality form with emphasis on surgical resection and radiotherapy [5]. Prognosis of HNC patients in post-treatment condition, however, is primarily the result of subjective analysis during treatment by assessment of histological grade, the pattern of tumour invasion, the proximity of carcinoma to resection margins and the presence of extra-nodal spread [5]. Therefore, it is observed that the current strategy for assessment of cancer prognosis in HNC is primarily qualitative and subjective. MicroRNA (miRNA) analysis offers an alternative method for assessment of prognosis which is both quantitative and objective. The deregulated miRNA expression profile found in the cancerous state of HNC has been suggested as an avenue for cancer screening, diagnosis and prognosis [6].

miRNAs that regulate the expression of genes involved in cancer development and progression may offer a non-invasive, sufficiently sensitive method for HNC detection, monitoring and prognosis [7]. It is now well recognised that aberrant expression of miRNA is connected with cancer development and progression [8]. A number of studies have also identified some miRNAs as having great potential as diagnostic and predictive biomarkers [9, 10]. A number of miRNA were also reported to be either over-expressed, under-expressed or dysregulated in HNC patients [11, 12]. However, despite significant methodological progress, concrete biomarkers capable of guiding treatment are yet to be identified. A comprehensive systematic review and meta-analysis consisting of data accumulated from a broad set of published studies will allow us to explore the implication of prognostic specific miRNA expression in HNC patient survival. Establishment and substantiation of specific miRNAs as reliable prognostic markers may also allow for treatment modalities in HNC to be patient-centric, leading to enhanced survival rates in patients. miRNA expression profiling of HNC patients will also serve to identify the expression rates of the prognostic specific miRNAs and will help predict treatment response in patients [13]. Thus, comparison of expression profiles between primary and metastatic HNC cancer patients may serve to underline miRNA that plays a major role as prognostic markers.

There is a dearth of comprehensive analyses verifying the effectiveness of miRNAs as prognostic biomarkers in HNC. Previous studies have explored prognostic effects of miRNAs in HNC patients, but the scope of these studies has been limited to a few miRNAs. This study will focus on previous clinical studies and investigate the significance of multiple miRNAs found to be under-expressed or over-expressed in HNC as viable prognostic markers. Furthermore, it will also put into perspective the over-representation of a few significant miRNAs observed in a majority of clinical studies investigating the prognostic utility of miRNA in HNC [14].

Though previous studies are investigating the prognostic utility of miRNA in HNC exist, they are limited to either a single miRNA or a small set of miRNA [14]. This approach limits the scope on the variety of miRNAs that could be used as prognostic biomarkers. This systematic review and meta-analysis of prognostic specific miRNA expression in HNC will ameliorate this issue by focusing on miRNA expression profiles as well as individual significantly expressed miRNA. This review will attempt to highlight patient clinical outcome patterns based on miRNA expression, which will inform clinical decision makers on patient-centric HNC treatment and management.

### Objectives

1. To identify the specific miRNAs involved in head and neck cancer prognosis.
2. To investigate the overall effect of miRNA expression on prognostic outcomes in patients with head and neck cancers.
3. To establish the up- and downregulated miRNAs in head and neck cancer metastasis.
4. To compare the miRNA profiles between metastatic and primary head and neck cancers.

## Methods

### The aim of the study

This study aims to analyse the outcomes of multiple previously published studies to evaluate the effectiveness of using miRNA as a reliable prognostic indicator in HNC. The pooled data will include studies focusing on multiple population cohorts and the survival outcomes of the HNC patients under different miRNA expression rates. The study will follow the PRISMA guidelines for systematic review and meta-analysis.

### Review questions

1. What are the significantly expressed miRNA in HNC?
2. What is the significance of miRNAs in determining patient survival?
3. Which miRNAs can potentially have utility as prognostic biomarkers in HNC?

### Study design

#### Search methods for identification of studies

The search strategy involves searching MEDLINE, Scopus, PubMed, Web of Science, Embase and Science Direct for published studies. The search strategy is designed to be broad and inclusive, with a prime focus on minimising bias while maximising sensitivity. This is to be achieved by utilising a broad set of keywords, which will allow for a comprehensive search (Table 1). Specific keywords will also contain subsets for better accuracy during the search. The keywords will be arranged in a strict order of relevance to study, to form a core search string, which will then be refined by the addition of combinations of the remaining keywords. To identify all relevant articles, the results of the primary search will be complemented with results obtained from Google Scholar, conference proceedings and published theses. The reference lists of screened articles will also be used to validate the robustness of the search strategy.

**Table 1:** Sample syntax for database search strategy

1.	“miRNA” [Topic] AND “Head and Neck Cancer” [Topic]
2.	“Head and Neck Cancer” [Topic] AND “miRNA” [Topic] AND “Prognosis” [Topic]
3.	“Head and Neck Cancer” [Topic] AND “Overall Survival” [Topic] OR “Disease Free Survival” [Topic] OR “Disease-Specific Survival” [Topic]
4.	“Head and Neck Cancer” [Topic] AND “miRNA expression” [Topic]
5.	“Head and Neck Cancer” [Topic] AND “miRNA” [Topic] AND “Upregulation” [Topic]
6.	“Head and Neck Cancer” [Topic] AND “miRNA” [Topic] AND “Downregulation” [Topic]
7.	“Head and Neck Cancer” [Topic] AND “miRNA” [Topic] AND “Deregulation” [Topic]
8.	“Head and Neck Cancer” [Topic] AND “miRNA” [Topic] AND “Biomarkers” [Topic]
9.	“Head and Neck Cancer” [Topic] AND “miRNA” [Topic] AND “Surgery” [Topic]
10.	“Head and Neck Cancer” [Topic] AND “miRNA” [Topic] AND “Radiotherapy” [Topic]
11.	“Head and Neck Cancer” [Topic] AND “miRNA” [Topic] AND “Chemotherapy” [Topic]
12.	“Head and Neck Cancer” [Topic] AND “miRNA” [Topic] AND “Clinical study” [Topic]
13.	“Oral cancer” [Topic] AND “miRNA” [Topic]
14.	“Head and Neck Squamous Cell Carcinoma” [Topic] AND “miRNA” [Topic]

The prospective keywords using Medical Subject Headings (MeSH) to be utilised in this search strategy, to form the core search, in order of importance, are:

1. miRNA
2. Head and Neck Cancer
3. Prognosis
4. Survival
  Overall Survival
  Disease-Free Survival
  Disease-Specific Survival
5. miRNA expression
  upregulation
  downregulation
  deregulation/dysregulation
6. Biomarkers
7. Treatment
  Surgical resection
  Radiotherapy
  Chemotherapy
8. Clinical study
9. Oral cancer
10. Head and Neck Squamous Cell Carcinoma (HNSCC)

#### Selection of studies

The studies will initially be selected based on the individual judgement of two authors upon reading of the title and abstract of the articles. Both prospective and retrospective observational studies involving miRNA expression assessment in HNC patients will be considered. Observational studies presenting study-specific data (e.g., hazard ratio, 95% confidence intervals) or sufficient data for an outcome measure to be calculated will also be included. There will be no restriction by study setting. Once relevant articles are screened in, a complete analysis of the full-text articles will be performed, by the previously defined selection criteria, independently by two authors. The authors will mark the articles as included, excluded or pending (if uncertainty about the inclusivity of the article arises). Any discrepancy will be resolved by discussion amongst the two authors. Any major disagreements will involve a team decision or third reviewer to generate a resolution. A flow chart describing this process will be generated to facilitate transparency.

### Criteria for considering studies for the review

The criteria for considering studies for the review are designed to be broad and inclusive. Any clinical study investigating miRNA expression in human participants or fresh/preserved tissue samples and correlating it with patient survival will be considered for the systematic review and meta-analysis. The broad criteria circumvent the issue of a small pool of published literature on the subject, which is often a major obstacle faced during systematic reviews and meta-analyses.

### Inclusion criteria

- Research studies that analyse miRNA expression in HNC patients
- Articles that investigate the association between miRNA expression and patient survival
- Studies that discuss validated miRNA screening in HNC
- Studies that discuss the clinicopathological characteristics of HNC patients
- Studies that are appropriate to the PRISMA guidelines for systematic review and meta-analysis
- Articles will be included, irrespective of any language of publication

### Participants

The systematic review and meta-analysis will include studies involving patients suffering from all types of HNC. Participants with clearly confirmed diagnoses of HNC will be included. To allow this study to be globally applicable, regardless of patient type, no restrictions will be placed on age, gender, region or ethnicity of the patients included in the study.

### Types of studies to be excluded

- Letters to the editor, case reports, case studies and review articles of HNC
- In vivo and in vitro studies of miRNA expression
- Studies investigating a patient/population cohort that has been represented previously in another study
- Studies involving patients suffering from oesophageal cancer as part of patient cohort representing HNC
- Self-reporting of the disease and questionable survey and screening methods of deduction have been employed.

#### Data extraction and management

The HNC miRNA prognosis literature will be stored into a reference management software EndNote™. This will contribute to a healthy working relationship among the review team during the study selection process. The authors will select the studies based on the study selection criteria and will upload relevant studies into EndNote™. This will assist in preparing a PRISMA flow diagram after the screening process by the HNC miRNA prognosis review team. HNC reviewers will also be using the traditional forms of data management in this process. A standardised, pre-piloted form will be used to extract data from each eligible study for assessment of study quality and data synthesis. Text, tables and figures from these studies will be used to extract the required data. All extracted data will be converted to Microsoft Excel spreadsheets for ease of data handling.

##### Data items

The standardised data extraction form for this study will contain the following parameters:

- Name of the first author
- Year of publication
- Country
- Number of participants
- Study population
- Assay methods
- Tumour stage
- Tumour anatomic location
- Clinicopathological characteristics (age, gender, risk factors and metastasis)
- Significantly expressed miRNAs
- Upregulated, downregulated and dysregulated miRNAs
- Hazard ratio (HR) with 95% confidence interval (CI) of patient survival including, overall survival (OS), disease-free survival (DFS) and disease-specific survival (DSS)

#### Assessment of risk of bias

Quality appraisal of selected articles will be done via a quality assessment tool developed by the National Heart, Lung, and Blood Institute (NHLBI) for observational and cross-sectional studies [15]. This assessment tool will be applied to all the selected full-text articles which will be rated as good, fair or poor. The risk of bias is inversely correlated with the quality of the study, with a high risk of bias translating to a rating of poor quality while a low risk of bias translates to a rating of good quality. Any disagreements between reviewers, during quality appraisal, will be resolved by the involvement of a third reviewer.

#### Dealing with missing data

Studies containing missing data will only be included if the missing data is procured by contacting the corresponding authors of such studies. If not, the studies containing partial data will be rejected from inclusion in the systematic review and meta-analysis to maintain the quality of the study.

#### Assessment of reporting bias

Publication bias will be evaluated using the Egger’s bias indicator test. The symmetry of funnel plots generated using log [HR] and standard error (S.E.) will also be used to assess for publication bias visually.

#### Data synthesis

Meta-analysis uses an aggregate of quantitative synthesis of various miRNA studies and yields mean effect size of the prognostic effects of miRNAs to enhance the power and reduce inconsistency. The effect size is the HR that provides the relative risk for survival data.

The pooled HR values from selected studies will be used to obtain a summary estimate of the relationship between miRNA expression and survival. Comprehensive Meta-Analysis (CMA) software will be used to analyse the data and perform a subsequent meta-analysis. Forest plots will be constructed using subsets from these total studies and will also be used for subgroup analysis for individual miRNA. The pooled HR and standard errors (95% CI values) will also be generated and reported.

We will manage a combination of unadjusted and adjusted hazard ratio estimates for the association between survival and the prognostic factor by using the patient-level correlation itself as an approximation for the within-study correlation [16]. The alternative ‘overall correlation’ multivariate model [17] can again be fitted without within-study correlations.

The common challenges are poor reporting of survival data, multiple populations and variation across the studies and publication bias. The studies in the analysis will be sampled from a universe of possible studies defined by our inclusion and exclusion rules and PRISMA guidelines outlined in this protocol. For this reason, the random effects model will be employed for the analysis.

Meta-analysis of prognostic factor studies often encounters multiple cut-points and/or methods of measurement. Riley et al. [16] demonstrated two models of meta-analysis, such as a 95% prediction interval with a random-effects meta-analysis ideal for estimating the distribution of a factor’s prognostic effect across the different cut-points [18] and measurement methods and multivariate meta-analysis models for investigating multiple prognostic results for each factor, relating to different cut-points and/or methods of measurement [19].

#### Assessment of heterogeneity

Heterogeneity between the studies will be assessed using the *I*^2^ statistic, where an *I*^2^ value higher than 50% is considered indicative of substantial heterogeneity. A random or fixed effects model will be applied to the meta-analysis, based on heterogeneity. A *P* value of < 0.01 will be considered statistically significant for *Q* test. The *z*-test will also be included in the meta-analysis to indicate the number of standard deviations from the study mean that each study may deviate. Heterogeneity is determined by Eggers bias indicator test [20]. Quality assessment and statistical analysis would be performed [15]. CMA is used to determine the pooled HR and 95% CI [21].

#### Subgroup analysis

Subgroup analysis will only be performed if sufficient clinical data is available. The primary focus will be on the involvement of age and various risk factors associated with HNC on patient survival and their influence and effect upon specific miRNA prognostic markers and their expression. Further analysis may be performed based on different regional as well as ethnic subgroups. miRNAs have been expressed into low (downregulated), high (upregulated) and differential (dysregulated) levels. Subgroup analysis will be performed on all different miRNAs by three different expressions. Different expressions of the miRNAs and its associations with HNC survival will be measured.

#### Meta-regression

The heterogeneity could arise by not only technological platforms used, but also microRNAs variability expression, the number of microRNAs screened and characteristics of sample study. The source of heterogeneity will be estimated using meta-regression analysis of fitting covariates. The heterogeneity of proportional contributions of miRNA expression and survival outcomes with study covariates will be assessed using meta-regression analysis. The impact of proportional contributions of risk factor and combination of risk factors on fitting covariates such as technological platforms used, microRNAs variability expression, the number of microRNAs screened and characteristics of sample study will be calculated using meta-regression model.

#### Reporting of the review

The findings in the systematic review and meta-analysis will be summarised in a flow diagram that will outline the selection process as per PRISMA guidelines (2015 Statement) for reporting systematic reviews and meta-analysis [22]. This will include the list of excluded studies and the reasons for exclusion. In-text descriptions will be used to describe the qualitative data in the studies**.**

## Discussion

Previous reports have highlighted the association between miRNA expression and HNC disease prognosis. However, the key focus of a large number of those published studies is predominantly on a small subset of miRNAs, despite there being a large number of miRNAs that are upregulated or downregulated in HNC. Many of them have very little to no studies exploring their significance in the prognosis of HNC patients. This trend is also reflected in recent systematic reviews and meta-analysis which tend to focus on the prognostic effects of a small limited set of miRNAs. This proposed study will build upon previous studies in the field by highlighting the significant miRNAs in prognosis while also exploring the prognostic effects of the lesser studied miRNAs. The search strategy is designed, with no filters being placed on the type of miRNA to include all possible types of miRNAs in this study. The aim is to generate a comprehensive list of studies that acts as a sample set mirroring the current research landscape, with regard to the miRNA being considered as significant prognostic markers for HNC.

We believe that this study will build upon previously existing studies while simultaneously acting as a guide for future research into the prognostic effects of miRNAs in HNC, especially in miRNAs that are yet to be thoroughly investigated. Though studies relating to all types of miRNAs expressed in HNC may be sparse in the clinical format, some in vitro and in vivo studies have identified multiple miRNA targets, capable of being used as prognostic markers, indicating a possible increase in research about this subject shortly. This is an active field of prognostic cancer research, and as the literature expands, we shall continue to add and substantiate our systematic review and meta-analysis with new findings, by repeating searches and refining the review. In the interim, we put forward this protocol to disseminate the process of our review, therefore maintaining transparency and providing a guidepost to others attempting a similar line of enquiry for their reviews and analyses.

## Abbreviations

DFS: Disease-free survival
DSS: Disease-specific survival
HNC: Head and neck cancer
miRNA: MicroRNA
OS: Overall survival
